# Chemical Vapor Deposition of Uniform and Large-Domain Molybdenum Disulfide Crystals on Glass/Al_2_O_3_ Substrates

**DOI:** 10.3390/nano12152719

**Published:** 2022-08-07

**Authors:** Qingguo Gao, Jie Lu, Simin Chen, Lvcheng Chen, Zhequan Xu, Dexi Lin, Songyi Xu, Ping Liu, Xueao Zhang, Weiwei Cai, Chongfu Zhang

**Affiliations:** 1 School of Electronic Information, University of Electronic Science and Technology of China Zhongshan Institute, Zhongshan 528402, China; 2 College of Physical Science and Technology, Xiamen University, Xiamen 361005, China; 3 School of Information and Communication Engineering, University of Electronic Science and Technology of China, Chengdu 611731, China

**Keywords:** MoS_2_, chemical vapor deposition, substrate, glass

## Abstract

Two-dimensional molybdenum disulfide (MoS_2_) has attracted significant attention for next-generation electronics, flexible devices, and optical applications. Chemical vapor deposition is the most promising route for the production of large-scale, high-quality MoS_2_ films. Recently, the chemical vapor deposition of MoS_2_ films on soda-lime glass has attracted great attention due to its low cost, fast growth, and large domain size. Typically, a piece of Mo foil or graphite needs to be used as a buffer layer between the glass substrates and the CVD system to prevent the glass substrates from being fragmented. In this study, a novel method was developed for synthesizing MoS_2_ on glass substrates. Inert Al_2_O_3_ was used as the buffer layer and high-quality, uniform, triangular monolayer MoS_2_ crystals with domain sizes larger than 400 μm were obtained. To demonstrate the advantages of glass/Al_2_O_3_ substrates, a direct comparison of CVD MoS_2_ on glass/Mo and glass/Al_2_O_3_ substrates was performed. When Mo foil was used as the buffer layer, serried small bilayer islands and bright core centers could be observed on the MoS_2_ domains at the center and edges of glass substrates. As a control, uniform MoS_2_ crystals were obtained when Al_2_O_3_ was used as the buffer layer, both at the center and the edge of glass substrates. Raman and PL spectra were further characterized to show the merit of glass/Al_2_O_3_ substrates. In addition, the thickness of MoS_2_ domains was confirmed by an atomic force microscope and the uniformity of MoS_2_ domains was verified by Raman mapping. This work provides a novel method for CVD MoS_2_ growth on soda-lime glass and is helpful in realizing commercial applications of MoS_2_.

## 1. Introduction

Two-dimensional transition metal dichalcogenides materials, specifically molybdenum disulfide (MoS_2_), have emerged as an extremely important candidate for low-power, high-performance, and flexible electronics [1,2,3,4,5,6,7]. In order to achieve industry applications, batch production of high-quality and large-scale MoS_2_ films at low cost has become a major requirement. Chemical vapor deposition (CVD) has demonstrated great potential in the large-scale production of high-quality MoS_2_ films [8,9,10,11]. At present, CVD growth of large-area MoS_2_ continuous films larger than 4 inches and the deposition of single MoS_2_ domains with sizes up to the millimeter-scale have been realized by independent research groups [8,9,10,12,13,14,15], which greatly promote the industrialization process of MoS_2_. Although those encouraging advancements have been made in the CVD growth of MoS_2_, related studies are still at an early stage for the industrialization of MoS_2_ films [16,17,18]. For example, the size of CVD-grown, single-crystalline graphene has reached up to the meter-scale with very fast growth rates [19,20]. However, there is an obvious gap between CVD MoS_2_ and graphene both in crystal size and quality [14,15]. More efforts should be made on designing a new growth set-up, searching for possible catalysts and promoters, testing appropriate growth substrates and carefully optimizing the CVD growth parameters to lower the cost and improve the quality and uniformity of CVD MoS_2_ [18,21].

Recently, low-cost soda-lime glass has been utilized as a growth substrate for CVD MoS_2_ growth [12,22,23,24,25,26,27,28]. In 2017, Chen et al. synthesized large-size MoS_2_ crystals on molten glass at 1050 °C [27]. In 2018, Gao et al. synthesized high-quality bilayer MoS_2_ with domain sizes up to 200 μm on molten glass at 830 °C [25]. Zhang et al. reported single-crystal monolayer MoS_2_ grown on molten glass at 850 °C with a domain size larger than 500 μm [24]. Yang et al. from Peking University developed a face-to-face metal precursor supply approach and deposited 6-inch uniform monolayer MoS_2_ on the glass [12]. In 2020, Zeng et al. demonstrated bandgap tuning of MoS_2_ grown on molten glass by a sphere diameter engineering technique [23]. In 2022, Li et al. reported the evolution of crystalline morphology of MoS_2_ grown on glass substrates and provided an effective approach to engineering the morphology of MoS_2_ crystals [22]. Commonly, due to the growth temperature of MoS_2_ being higher than soda-lime glass’s melting point, a piece of Mo foil or graphite needed to be used as a buffer layer between the glass substrates and the CVD system in those works to prevent the adhesion of glass substrates and CVD systems [25,26,27]. However, the MoS_2_ morphology and domain size may be critically affected by Mo foil because Mo foil also could be used as the Mo-source in the chemical vapor deposition process [26]. In addition, Mo foils and graphite are very easy to react with oxygen at high growth temperatures, which limits the research approaches to MoS_2_ growth on glass substrates, although oxygen has been proven to be an effective gas for improving the size and quality of MoS_2_ crystals [8,9,29,30,31,32]. For example, Zhang’s group obtained a 4-inch monolayer MoS_2_ film on a sapphire substrate by epitaxial growth using an oxygen-assisted method [13]. Hence, for the aim of Mo-source precise control and to extend the capability of glass substrates for high-quality MoS_2_ CVD growth at variable critical experimental conditions, a non-active buffer layer needs to be explored.

In this work, high-quality CVD monolayer MoS_2_ films grown on glass/Al_2_O_3_ substrates were explored. Firstly, to demonstrate the advantages of glass/Al_2_O_3_ substrates, MoS_2_ films were synthesized on glass/Al_2_O_3_ and glass/Mo substrates and characterized with optical microscopy in different regions. At the center of the glass/Mo substrates, serried bilayer seeds could be observed on the monolayer MoS_2_ domains, while the MoS_2_ domains grown on glass/Al_2_O_3_ substrates exhibited a uniform contrast. At the edge of the glass/Al_2_O_3_ substrates, monolayer MoS_2_ domains with low nucleation density and uniform contrast were grown. As a control, monolayer MoS_2_ domains with high nucleation density and multi-layer nucleation sites were grown on the edge region of glass/Mo substrates. Subsequently, the grown MoS_2_ thin films were successfully transferred onto SiO_2_/Si substrates and characterized with Raman and photoluminescence (PL) spectroscopy, as well as atomic force microscopy (AFM). A comparison of Raman and PL spectra of MoS_2_ domains grown by those two methods further illustrates the advantages of the non-active buffer layer, where uniform MoS_2_ domains were synthesized as a result of a onefold supply of Mo-source. Finally, the thickness and uniformity of MoS_2_ domains on glass/Al_2_O_3_ substrates were confirmed by AFM and Raman mapping.

## 2. Experiments and Methods

**Figure 1:**
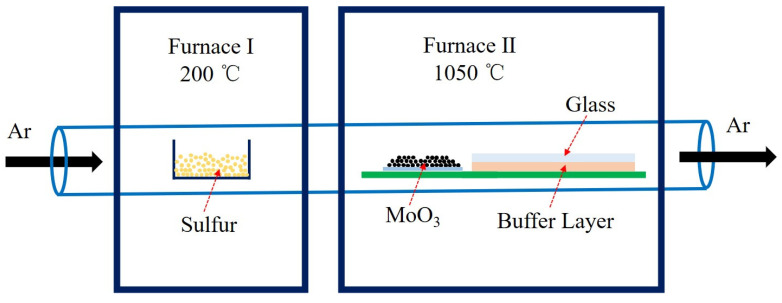
Schematic illustration of the CVD growth system setup.

As depicted in Figure 1, the MoS_2_ crystals were grown in a CVD system with a 2-inch-diameter quartz tube and two individual furnaces. In this system, the temperatures of substrates and precursors, carrier gas flow rate, total pressure, and the weight of precursors could be controlled independently. Following the general conditions reported in previous literature [24,25], 1.4 g of sulfur powder and 2 mg of MoO_3_ powder were weighed out and placed into separate boats. The boats were placed 25 cm apart in separate regions of the quartz tube to achieve the different temperatures for the S and Mo precursors. Both glass substrates and the buffer layers (Mo foils or Al_2_O_3_ lamina) were cleaned with acetone (10 min), isopropanol (10 min) and deionized water (10 min). The size of both the substrates and buffer layers was 20 mm × 20 mm. After loading precursors and substrates, the tube was first pumped down to 0.2 mBar and then filled with Ar to 1000 mBar. The pumping and filling processes were repeated three times to eliminate air and other containment gases in the quartz tube. After that, the temperatures of furnaces I and II were set to 200 and 1050 °C, respectively, with a ramping rate of 10 °C/min. During the growth, high-purity argon was loaded with a flow rate of 20 sccm. After a growth period of 10 min, the furnaces were naturally cooled to room temperature.

## 3. Results and Discussion

**Figure 2:**
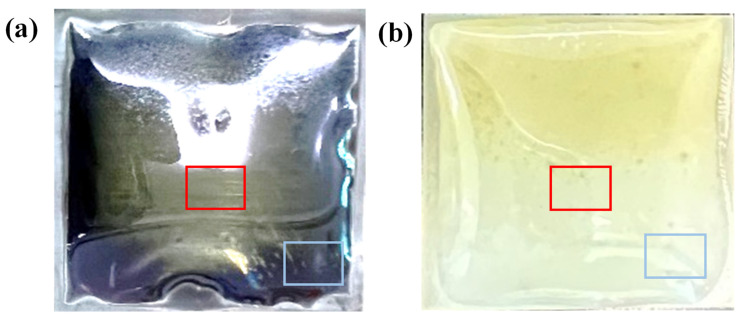
(**a**) Optical photograph of the glass/Mo substrates after the growth of MoS_2_. (**b**) Optical photograph of the glass/Al_2_O_3_ substrates after the growth of MoS_2_. The red rectangle corresponds to the center region, and the blue rectangle corresponds to the edge region.

Figure 2a,b are the optical photographs of glass/Mo and glass/Al_2_O_3_ substrates after the growth of MoS_2_, respectively. The Mo foil buffer layer displays a color of gray, and the Al_2_O_3_ buffer layer shows different a color of white. Furthermore, the glass substrates shrunk obviously, and the edge of Mo foil and Al_2_O_3_ lamina showed up after the high-temperature growth process [23,26]. As we all know, the shape, distribution, and thickness of MoS_2_ domains depend on the weight of precursors provided to the substrates. Consequently, as illustrated in Figure 2a,b, non-uniform MoS_2_ film growth could be observed in different regions due to the different precursors’ concentration distributions on the glass substrates [12,33,34]. Typically, in our growth setup, thicker continuous MoS_2_ films could be grown upstream, closer to the Mo-source. Individual monolayer MoS_2_ domains tend to be synthesized downstream, farther away from the Mo-source, and abundant growth phenomena could be observed in these regions. Therefore, in order to clearly demonstrate the advantages of glass/Al_2_O_3_ substrates, the MoS_2_ morphology on the center and edge regions, as shown in Figure 2, was analyzed.

**Figure 3:**
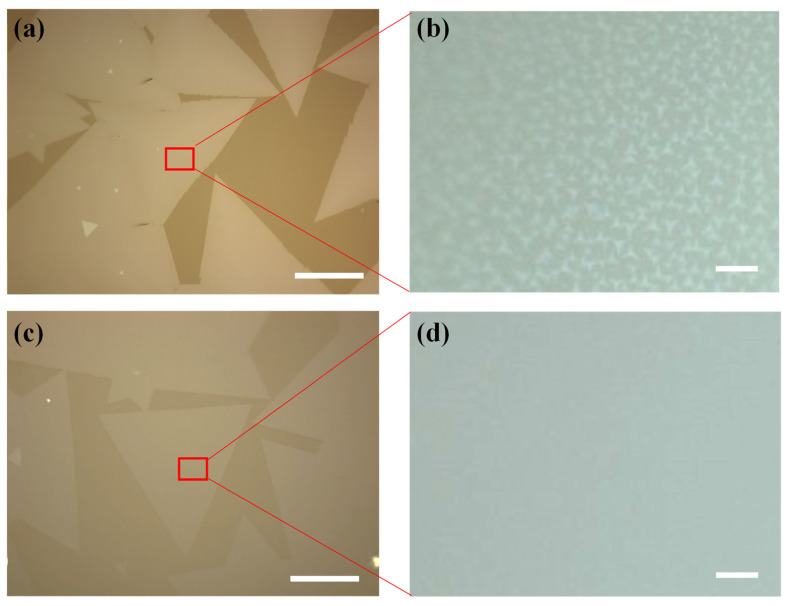
(**a**,**b**) Optical microscope images of the MoS_2_ domains synthesized on the center region of glass/Mo substrates. (**c**,**d**) Optical microscope images of the MoS_2_ domains synthesized on the center region of glass/Al_2_O_3_ substrates. Scale bar represents 200 μm for (**a**,**c**), and 10 μm for (**b**,**d**).

**Figure 4:**
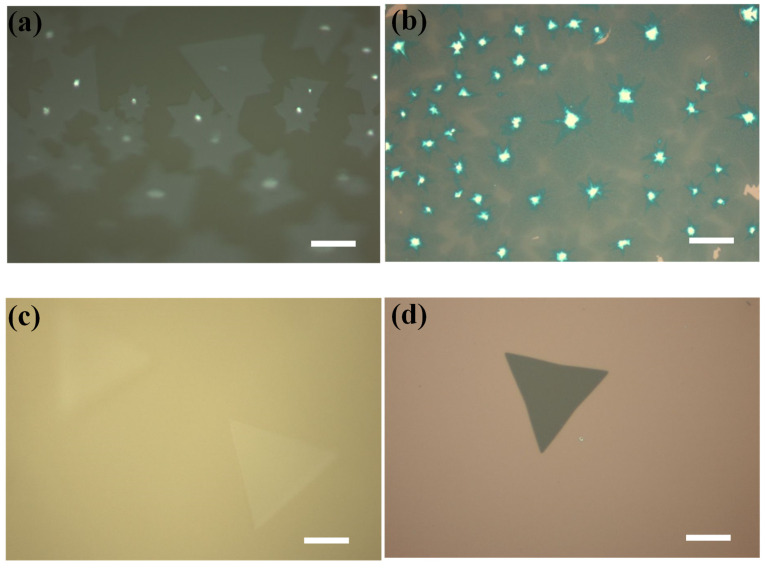
(**a**,**b**) Optical microscope images of the MoS_2_ domains synthesized on the edge region of glass/Mo substrates. (**c**,**d**) Optical microscope images of the MoS_2_ domains synthesized on the edge region of glass/Al_2_O_3_ substrates. Scale bar represents 50 μm for (**a**–**d**).

The MoS_2_ domains grown on different regions of glass/Al_2_O_3_ and glass/Mo substrates are illustrated in Figure 3 and Figure 4. Figure 3a,b are the typical optical microscopy images obtained from the MoS_2_ domains grown on the center region of glass/Mo substrates. The MoS_2_ domains exhibited triangle shapes and demonstrated lateral sizes larger than 400 μm, which is comparable to the MoS_2_ domain sizes on glass substrates reported in other works [12,26]. The large crystal size could be attributed to the smooth, molten surface together with the catalytic role of the glass substrates, as reported in previous works [12,26,27,35,36]. As shown in Figure 3b, numerous tiny islands could be observed on the large MoS_2_ triangle domains after additional magnification. The small bilayer islands could be attributed to the Mo foil buffer layer, which provides additional Mo-source during the growth of MoS_2_ domains. Figure 3c,d are the representative optical microscopy images obtained from the MoS_2_ domains grown on the center region of glass/Al_2_O_3_ substrates. Triangle MoS_2_ domains larger than 400 μm with uniform color contrast could be observed. In addition, no small bilayer islands could be observed on the amplified image of the MoS_2_ domain, which demonstrates the thickness uniformity of MoS_2_ crystal domains grown on the glass/Al_2_O_3_ substrates.

Figure 4 shows the MoS_2_ domains on the edge of the glass/Mo and the glass/Al_2_O_3_ substrates. As shown in Figure 4a, the MoS_2_ domains on the edge region of glass/Mo substrates generally exhibit star shapes with bright core centers. In contrast, the MoS_2_ domains on the edge region of glass/Al_2_O_3_ substrates are sparse and generally exhibit triangle shapes with uniform thickness, as shown in Figure 4c. In addition, both the MoS_2_ domains grown on glass/Mo and glass/Al_2_O_3_ were successfully transferred to SiO_2_/Si substrates. Figure 4b shows the transferred MoS_2_ domains with bright core centers that were grown on glass/Mo substrates. Figure 4d shows the transferred MoS_2_ domains with uniform thickness that were grown on glass/Al_2_O_3_ substrates. The details of the transfer method have been reported in our previous works [24,25,37,38].

**Figure 5:**
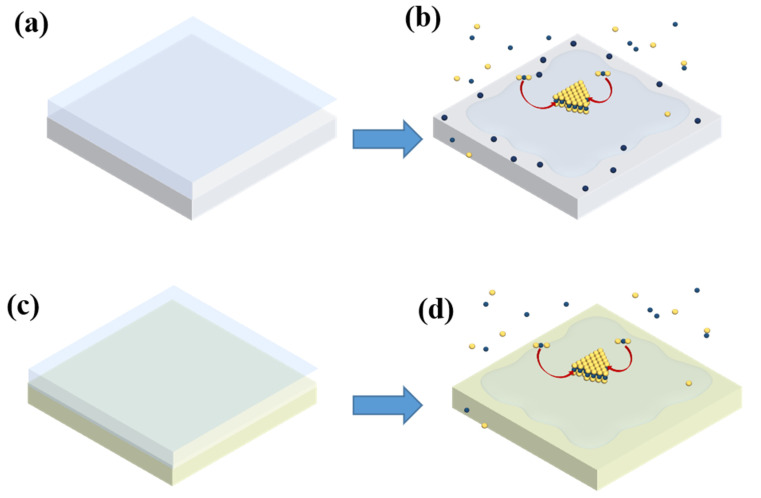
(**a**,**b**) Schematic illustration of the CVD growth process on glass/Mo substrates. (**c**,**d**) Schematic illustration of the CVD growth process on glass/Al_2_O_3_ substrates. The blue balls and yellow balls in (**b**,**d**) represent Mo and S atoms, respectively.

To provide a clear explanation of the different morphologies of the MoS_2_ domains grown on glass/Mo and glass/Al_2_O_3_ substrates, a schematic illustration of the underlying growth mechanism was plotted. Typically, as shown in Figure 5a,b, the flat glass plane will be condensed into an oblate, sphere-like shape at a temperature higher than its molten point [23]. Subsequently, the Mo foil or Al_2_O_3_ buffer layer under glass substrates would be exposed to the CVD system. The Mo foil could be used as the Mo-source in the high-temperature CVD process, which has been demonstrated in previous work [26]. Therefore, excess Mo-source would be supplied and diffused on the glass/Mo substrates’ surface. Consequently, the MoS_2_ domains with small bilayer islands and bright core centers were grown at the center and edge regions, respectively. On the contrary, the strong chemical bonds of Al_2_O_3_ make it stable at high temperatures. Therefore, as shown in Figure 5c,d, the Al_2_O_3_ buffer layer would not affect the Mo-source supply during the process of MoS_2_ growth and MoS_2_ crystals with uniform thickness were grown. Furthermore, as discussed in previous works [12,26,27,35,36], the large domain size of MoS_2_ at the center region of glass substrates could be ascribed to the smooth, molten glass surface under high temperature, together with the catalytic role of Na in the glass substrates, which reduce the energy barrier in the CVD process.

**Figure 6:**
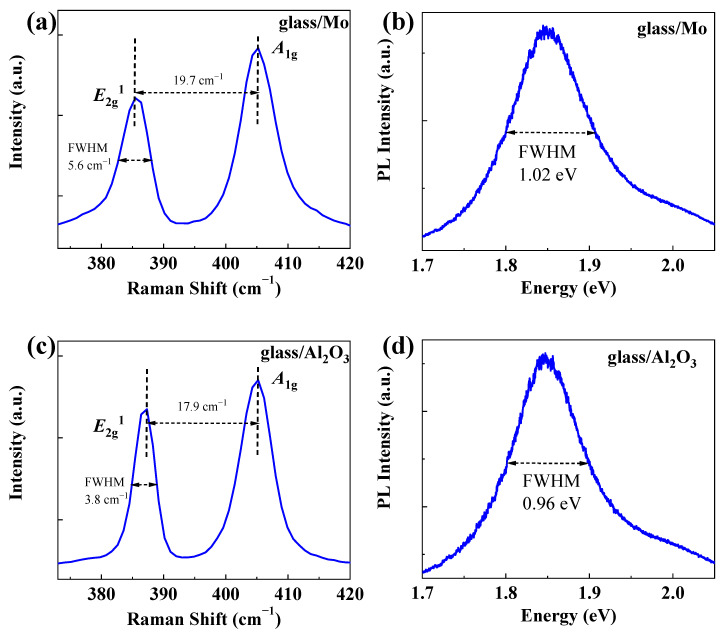
(**a**,**b**) Raman and PL spectral of the CVD-grown MoS_2_ on the center region of glass/Mo substrates. (**c**,**d**) Raman and PL spectral of the CVD-grown MoS_2_ on the center region of glass/Al_2_O_3_ substrates.

Raman spectroscopy is one commonly used spectroscopic technique to investigate phonons as well as the vibrational, rotational and other low-frequency modes in two-dimensional materials, which could be used to provide information about both crystal quality as well as estimate the number of layers of MoS_2_ domains [39,40,41,42]. Therefore, Raman spectra were collected to further characterize the properties of MoS_2_ grown on glass/Mo and glass/Al_2_O_3_. Figure 6a,c display the Raman spectra of the transferred MoS_2_ domains on SiO_2_/Si substrates that were grown on the center regions of glass/Mo and glass/Al_2_O_3_ substrates, respectively. As shown in Figure 6a, two characteristic Raman peaks were found at 385.4 cm^−1^ and 405.2 cm^−1^ in the spectral range, which can be assigned to in-plane vibration modes of Mo and S in the opposite direction (*E*^1^_2g_) and an out-of-plane vibration mode of S atoms (*A*_1g_). The frequency difference for the MoS_2_ domains grown on glass/Mo substrates was 19.7 cm^−1^. For the MoS_2_ domains grown on glass/Al_2_O_3_ substrates, the *E*^1^_2g_ and *A*_1g_ peaks are located at 387.3 cm^−1^ and 405.2 cm^−1^. Compared with the MoS_2_ domains grown on glass/Mo substrates, the frequency difference was reduced to 17.9 cm^−1^, indicating a monolayer thickness for the measured MoS_2_ domains. Moreover, the full width at half maximum (FWHM) of the *E*^1^_2g_ peak also reduced from 5.6 cm^−1^ to 3.8 cm^−1^, demonstrating the high quality of the MoS_2_ domains grown on glass/Al_2_O_3_ substrates. The reduced frequency difference and FWHM results from the different morphologies of MoS_2_ domains grown on glass/Mo and glass/Al_2_O_3_ substrates, as shown in Figure 3. In addition, PL spectroscopy was performed for the transferred MoS_2_ domains grown on glass/Mo and glass/Al_2_O_3_ substrates, and the obtained spectra are shown in Figure 6b,d. Both those two types of MoS_2_ have a characteristic peak (A exciton peak) at 1.84 eV, which is in agreement with previous studies [24,25]. The FWHMs of the A exciton peaks for MoS_2_ domains grown on glass/Mo and glass/Al_2_O_3_ substrates are 1.02 and 0.96 eV, respectively. The reduced PL FWHMs of MoS_2_ domains grown on glass/Al_2_O_3_ could also be attributed to its uniform thickness and high crystal quality.

**Figure 7:**
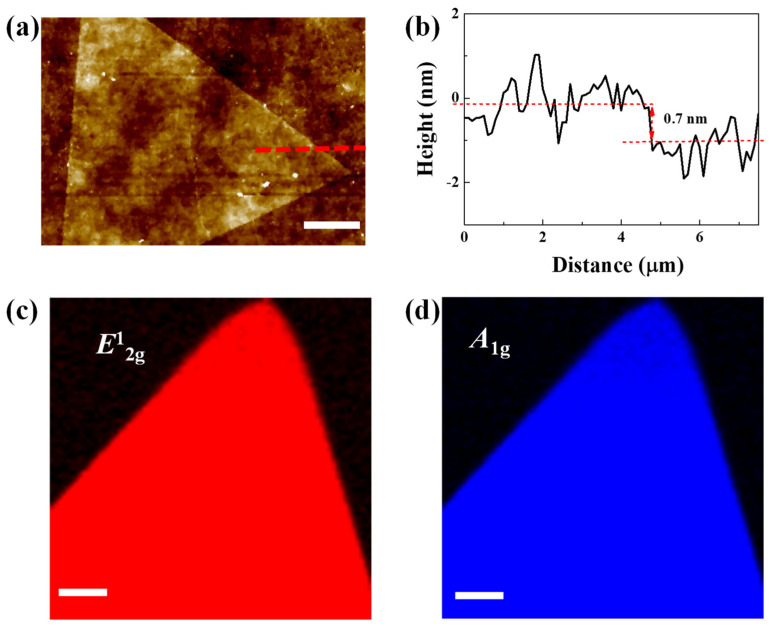
(**a**,**b**) AFM image and height profile of the CVD-grown MoS_2_ on glass/Al_2_O_3_ substrates. (**c**,**d**) Raman mapping of the CVD-grown MoS_2_ on glass/Al_2_O_3_ substrates. Scale bar are 5 μm for (**a**), and Scale bars are 3 μm for (**c**,**d**).

In order to further characterize the MoS_2_ domains grown on glass/Al_2_O_3_ substrates, atomic force microscopy (AFM) and Raman mapping were performed after they were transferred onto SiO_2_/Si substrates. Figure 7a displays the AFM images obtained from a typical triangle-shaped MoS_2_ domain. A thickness of 0.7 nm was demonstrated through the AFM characterization, as shown in Figure 7b. This is consistent with the thickness of the monolayer MoS_2_. Figure S1 displays the optical microscope and AFM images of the CVD-grown MoS_2_ on the center region of glass/Al_2_O_3_ substrates. As shown in Figure S1b, numerous tiny islands could be observed on the large MoS_2_ triangle domains. Figure 7c,d show the Raman intensity mappings recorded at 387.3 cm^−1^ and 405.2 cm^−1^, respectively. The Raman mappings on the intensity of *E*^1^_2g_ mode and *A*_1g_ mode reveal a uniform color contrast, further demonstrating the thickness uniformity and good crystallinity of MoS_2_ grown on glass/Al_2_O_3_ substrates.

## 4. Conclusions

In this work, the two-dimensional semiconductor MoS_2_ was successfully synthesized on glass/Al_2_O_3_ substrates for the first time. The advantages of glass/Al_2_O_3_ substrates for CVD MoS_2_ growth were demonstrated by a direct comparison of glass/Mo substrates. Optical microscopy revealed that MoS_2_ crystals grown on both glass/Mo and glass/Al_2_O_3_ substrates were conventionally triangle-shaped and had a lateral size larger than 400 μm. Numerous bilayer islands and bright core centers could be observed on the surface of MoS_2_ domains at the center region and edge region of glass/Mo substrates. As a control, MoS_2_ domains grown on glass/Al_2_O_3_ substrates exhibited uniform optical contrast, demonstrating the thickness uniformity of those MoS_2_ domains. The Raman and PL comparison of MoS_2_ domains further confirmed this. In addition, the thickness of MoS_2_ domains was characterized by atomic force microscopy and the homogeneity of MoS_2_ domains grown on glass/Al_2_O_3_ substrates was further demonstrated by Raman mapping. These results demonstrate a novel method to produce MoS_2_ films on glass substrates and are of great value for future commercial applications of MoS_2_ films.

## Data Availability

The data that support the findings of this study are available from the corresponding author upon reasonable request.

## Supplementary Materials

The following supporting information can be downloaded at: https://www.mdpi.com/article/10.3390/nano12152719/s1, Figure S1: AFM image of the CVD growth MoS_2_ on glass/Mo substrates.
