# Supplementary material for: Chemical Vapor Deposition of Uniform and Large-Domain Molybdenum Disulfide Crystals on Glass/Al2O3 Substrates
Source: Nanomaterials (Basel). 2022 Aug 7;12(15):2719. doi: 10.3390/nano12152719 (PMC9370393; doi:10.3390/nano12152719)
Supplement: Supplementary file 1 [file nanomaterials-12-02719-s001.zip › nanomaterials-1853238-supplementary.pdf]

nanomaterials

Supplementary Materials

Chemical Vapor Deposition of Uniform and Large-Domain Molybdenum Disulfide Crystals on Glass/Al_2O_3 Substrates

Qingguo Gao [1,*], Jie Lu [2], Simin Chen [1], Lvcheng Chen [1], Zhequan Xu [1], Dexi Lin [1], Songyi Xu [1], Ping Liu [1], Xueao Zhang [2], Weiwei Cai [2] and Chongfu Zhang [1,3]

[1] School of Electronic Information, University of Electronic Science and Technology of China Zhongshan Institute, Zhongshan 528402, China
[2] College of Physical Science and Technology, Xiamen University, Xiamen 361005, China
[3] School of Information and Communication Engineering, University of Electronic Science and Technology of China, Chengdu 611731, China
* Correspondence: gqgemw@163.com

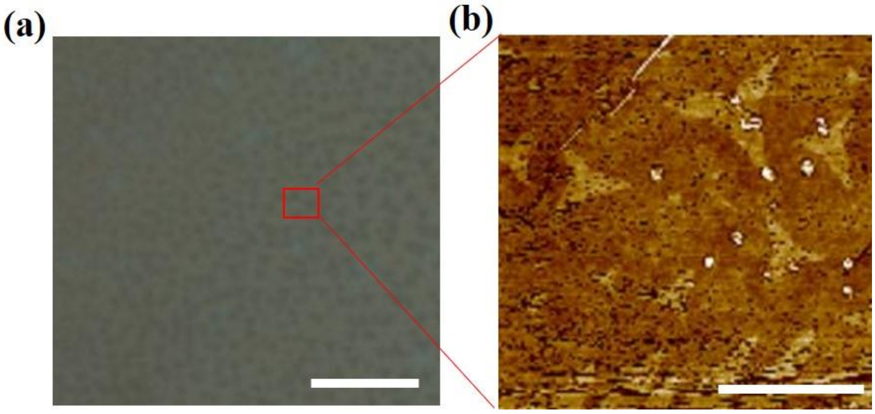

Figure S1. (**a**) Optical microscope images of the MoS_2 domains synthesised on the center region of glass/Mo substrates. The scale bar is 5 µm. (**b**) AFM image of the CVD growth MoS_2 on glass/Mo substrates. The scale bar is 1 µm.
